# Diagnosis Osteoporosis Risk: Using Machine Learning Algorithms Among Fasa Adults Cohort Study (FACS)

**DOI:** 10.1002/edm2.70023

**Published:** 2025-01-06

**Authors:** Saghar Tabib, Seyed Danial Alizadeh, Aref Andishgar, Babak Pezeshki, Omid Keshavarzian, Reza Tabrizi

**Affiliations:** ^1^ Student Research Committee, School of Medicine Fasa University of Medical Sciences Fasa Iran; ^2^ Sina Trauma and Surgery Research Centre Tehran University of Medical Sciences Tehran Iran; ^3^ Clinical Research Development Unit, Valiasr Hospital Fasa University of Medical Sciences Fasa Iran; ^4^ School of Medicine Shiraz University of Medical Sciences Shiraz Iran; ^5^ Noncommunicable Diseases Research Center Fasa University of Medical Sciences Fasa Iran; ^6^ USERN Office, Fasa University of Medical Sciences Fasa Iran

**Keywords:** diagnosis, Fasa Adult Cohort Study, machine learning, osteoporosis

## Abstract

**Introduction:**

In Iran, the assessment of osteoporosis through tools like dual‐energy X‐ray absorptiometry poses significant challenges due to their high costs and limited availability, particularly in small cities and rural areas. Our objective was to employ a variety of machine learning (ML) techniques to evaluate the accuracy and precision of each method, with the aim of identifying the most accurate pattern for diagnosing the osteoporosis risks.

**Methods:**

We analysed the data related to osteoporosis risk factors obtained from the Fasa Adults Cohort Study in eight ML methods, including logistic regression (LR), baseline LR, decision tree classifiers (DT), support vector classifiers (SVC), random forest classifiers (RF), linear discriminant analysis (LDA), K nearest neighbour classifiers (KNN) and extreme gradient boosting (XGB). For each algorithm, we calculated accuracy, precision, sensitivity, specificity, F1 score and area under the curve (AUC) and compared them.

**Results:**

The XGB model with an AUC of 0.78 (95% confidence interval [CI]: 0.74–0.82) and an accuracy of 0.79 (0.75–0.83) demonstrated the best performance, while AUC and accuracy values of RF were achieved (0.78 and 0.77), LR (0.78 and 0.77), LDA (0.78 and 0.76), DT (0.76 and 0.79), SVC (0.71 and 0.64), KNN (0.63 and 0.59) and baseline LR (0.72 and 0.82), respectively.

**Conclusion:**

The XGB model had the best performance in assessing the risk of osteoporosis in the Iranian population. Given the disadvantages and challenges associated with traditional osteoporosis diagnostic tools, the implementation of ML algorithms for the early identification of individuals with osteoporosis can lead to a significant reduction in morbidity and mortality related to this condition. This advancement not only alleviates the substantial financial burden placed on the healthcare systems of various countries due to the treatment of complications arising from osteoporosis but also encourages health policies to shift toward more preventive approaches for managing this disease.

## Graphical abstract

We entered 150 variables in various fields into this study and using the permutation importance technique and RF model, we identified the most important features. We trained the models using balanced data and then evaluated and compared the performance of the models in different areas. Ultimately, the XGB was selected as the best model for diagnosis osteoporosis risk.

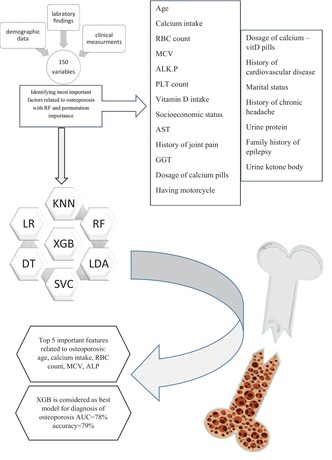

**Summary**

- This study applied machine learning to identify risk factors for osteoporosis, aiming to create an early detection tool for high‐risk individuals in Iran. The extreme gradient boosting model showed the highest accuracy and effectiveness, highlighting age, calcium intake and red blood cell count as key diagnoses in osteoporosis risk assessment.

## 1 Introduction

Osteoporosis is the most common bone disease in humans, indicating a major public health issue that results in reduced bone density and tissue destruction [1, 2, 3]. It is estimated that in developed countries, there are about 49 million people suffering from osteoporosis [4]. Based on data from the National Institute of Health and Nutrition in the United States, the prevalence of osteoporosis is approximately 19.6% in women and 4.4% in men over 50 years old between 2017 and 2018 [5], and approximately one‐third of Iranians over the age of 60 suffer from osteoporosis [6]. Considering the increasing number of cases and the uncertain treatments, this disease has become a significant issue in the field of healthcare and expresses the importance of early diagnosis, screening and prevention [2, 4, 7]. Given the increased life expectancy and longevity of individuals in most countries today, along with the significant rise in age‐related diseases such as osteoporosis, determining efficient and cost‐effective methods for identification and screening has become of great importance.

World Health Organization (WHO) diagnostic criteria for osteoporosis include measuring bone mineral density (BMD) based on a dual X‐ray absorptiometry (DEXA) scan [8, 9]. However, many fractures caused by osteoporosis occur with BMD at borderline or even normal levels, raising questions about the sensitivity of this method, particularly for screening purposes [9]. Although DEXA Scan is widely accepted as the gold standard diagnostic tool for this disease, due to the high cost of use in a large population, numerous side effects and impracticality, it is not useful for screening and early detection of high‐risk individuals. The high cost of purchasing and maintaining this device renders its use impractical in many healthcare centers in many developing countries [8, 10, 11]. Alternative methods have recently been developed to evaluate BMD levels, such as quantitative computed tomography and radiographic absorptiometry. However, the high costs of acquiring and maintaining necessary equipment pose significant challenges to the use of these alternative methods, particularly in developing countries with weak economies. This renders the widespread use of these methods as general screening tools impractical, particularly in developing countries where budget constraints and other issues often result in the lack of basic health services [10]. For example, DEXA scans are accessible to the public in major research and treatment centers in developing countries such as Morocco and Egypt; however, in countries like India and sub‐Saharan Africa, these tools are only available in major cities and private hospitals. Therefore, the use of these tools is limited to wealthy and privileged individuals [12]. So, choosing a high‐sensitivity, cost‐effective and accessible tool for screening is of great importance.

Machine learning (ML) is a subset of artificial intelligence that can analyse different variables in multiple layers and compare the significance of each variable as a risk factor for developing the specific disease [5]. Compared to other statistical methods, ML places more emphasis on the accuracy of predictions and diagnoses and can recognise patterns in the data provided to it [13]. ML algorithms have recently been utilised in a variety of research studies for the prediction and early detection of numerous diseases, particularly chronic conditions such as osteoporosis [5].

The effective application of ML algorithms for patient screening and identification necessitates comprehensive research and expert evaluations. In countries like Iran, where there is a critical need to harness the significant potential of this technology in clinical settings, we have undertaken this study to advance understanding and implementation in this area.

In this article, we aimed to analyse variables related to osteoporosis risk factors using data from the Fasa Adults Cohort Study (FACS). We employed a variety of ML techniques to evaluate the accuracy and precision of each method, aiming to identify the most accurate pattern for assessing osteoporosis risk and identifying individuals at risk. This study explores the use of ML algorithms as a cost‐effective, publicly accessible and highly accurate tool for identifying and screening individuals at risk of disease complications. This aligns with enhancing the efficacy of health policies that prioritise early diagnosis of diseases like osteoporosis, thereby preventing the numerous complications that may arise if the disease remains undiagnosed until its final stages.

## 2 Materials and Methods

### 2.1 Data Source

This study is a retrospective longitudinal study that utilises data from the FACS. The FACS study consists of 10,118 people, with 4566 men and 5552 women, all aged between 35 and 70 years, between October 2014 and September 2016. The purpose of FACS is to evaluate the risk factors that make Fasa's rural population more susceptible to noncommunicable diseases, including osteoporosis.

### 2.2 Study Population

This study included all participants with osteoporosis by convenience sampling. The participants with the following secondary diseases that could affect BMD, such as fracture, spinal tumour, spondylopathy and systemic disease, were excluded from the study. A total of 10,108 participants were analysed in the current study.

### 2.3 Data Pre‐Processing

All 150 variables used in the analysis had less than 15% missing data. For handling the missing data, we applied mean imputation for continuous variables and median imputation for categorical variables to ensure a comprehensive dataset for the ML models.

One‐hot encoding and feature scaling are essential preprocessing steps in ML. One‐hot encoding transforms categorical variables into a binary vector, where each category is represented by a unique bit set to 1, while the rest are set to 0, enabling algorithms to process categorical data effectively. Feature scaling, on the other hand, standardises numerical features to a common scale, typically using techniques such as min‐max scaling or z‐score normalisation. The min‐max scaling technique was implemented for this study. This ensures that each feature contributes equally to the model's performance, preventing features with larger scales from dominating the learning process. In the article, these techniques were implemented to ensure consistent and efficient training of the ML model by normalising data and enabling the algorithm to interpret categorical inputs correctly.

The FACS study identified individuals who were diagnosed with osteoporosis as having a *T*‐score of −2.5 or below, based on the criteria established by the WHO and the International Society for Clinical Densitometry (ISCD). These individuals were post‐menopausal women and men aged 50 and above. The ISCD recommends a *Z*‐score of −2.5 D or lower for diagnosing osteoporosis in people under the age of 50 [14].

### 2.4 ML Algorithm

This study involved training eight ML models to diagnose osteoporosis. Hyperparameter tuning was employed to identify the optimal parameters. To mitigate overfitting, the dataset was partitioned into two subsets: 80% for training and 20% for testing. The training process involved training the models, optimising hyperparameters and performing fivefold cross‐validation. The test data were independent of the training data and were utilised for the ultimate assessment and internal validation of the ML models.

The data were analysed using Python version 3.9.12. A total of 150 variables were collected from each patient, including demographic data, clinical measurements and laboratory results.

The models included logistic regression (LR), baseline LR, decision tree (DT), support vector classifier (SVC), random forest (RF), linear discriminant analysis (LDA), K nearest neighbours (KNN) and extreme gradient boosting (XGB). Each algorithm was selected for its distinct strengths in handling various data types and complexities. LR was chosen for its simplicity and interpretability in binary classification tasks. DT and RF were included for their ability to handle non‐linear relationships and their effectiveness in feature selection. SVC was used for its capacity to classify data in higher dimensions and its robustness to overfitting. LDA was selected for its suitability in situations where the classes are separable by linear boundaries. KNN was included for its simplicity and effectiveness in smaller datasets with well‐defined class boundaries. XGB was chosen for its superior performance in handling large datasets and complex relationships through its boosted ensemble approach. Finally, all the features were entered into the logistic regression model without selection to establish a basis for comparing results, and we used the results obtained from this baseline LR as a reference.

Furthermore, the Scikit‐Learn Module (Version 1.1.3) was utilised to execute the machine algorithms. Ultimately, two participants, one with osteoporosis and one without, were separated from the study population, and their Shapley Additive Explanations (SHAP) force plot was demonstrated.

### 2.5 Model Development and Evaluation

Initially, the training data underwent fivefold cross‐validation and hyperparameter tuning to determine the most optimal hyperparameters. All features were utilised at this level. The fivefold technique divided the training data into 5 equal parts. Each part was used as validation data once, while the rest were used for training. The accuracy was reported for each iteration, and the average of all 5 accuracies was calculated. The accuracy of each ML model can be modified by altering its hyperparameters. Different combinations of hyperparameters were used in the process of tuning hyperparameters to identify the optimal combination. The grid search approach was used for hyperparameter tweaking. The optimal hyperparameters are presented in Table S1. Additionally, the technique of oversampling was utilised to equalise the distribution of outcome values. The synthetic minority oversampling technique (SMOTE) was employed to equalise the distribution of the training data. This strategy employs oversampling to artificially increase the representation of the minority group by generating additional cases. The SMOTE algorithm chooses instances from the underrepresented class and generates synthetic instances along the same line segment, connecting some or all of the k nearest neighbours of the underrepresented class. The test data were subjected to all trained ML models. The evaluation metrics are determined using the following equations:

$$\text{Accuracy}=\left(\mathrm{TP}+\mathrm{TN}\right)/\left(\mathrm{TP}+\mathrm{FP}+\mathrm{TN}+\mathrm{FN}\right)$$

$$\text{Sensitivity}=\mathrm{TP}/\left(\mathrm{TP}+\mathrm{FN}\right)$$

$$\text{Specificity}=\mathrm{TN}/\left(\mathrm{TN}+\mathrm{FP}\right)$$

$$\text{Precision}=\mathrm{TP}/\left(\mathrm{TP}+\mathrm{FP}\right)$$

$$\text{F1 score}=2\times \mathrm{TP}/\left(2\times \mathrm{TP}+\mathrm{FP}+\mathrm{FN}\right)$$

TP stands for true‐positive rate, TN is for true‐negative rate, FP stands for false‐positive rate and FN stands for false‐negative rate.

### 2.6 Feature Selection

In diagnosis modelling, selecting the most relevant features is crucial for enhancing the model's performance and reducing computational complexity. Feature selection helps focus on the variables that have the greatest impact on the outcome, improving model efficiency and interpretability. Also, the baseline logistic regression model was used without feature selection to evaluate the effectiveness of this technique. Permutation importance with an RF classifier was used to select the top among 151 features to contribute to the diagnosis of osteoporosis. Permutation importance is a method used to evaluate the significance of features in an RF model. By randomly permuting the values of a feature and measuring the impact on the model's performance, this technique assesses how much the feature contributes to diagnosis accuracy. When a feature's values are shuffled, a significant drop in performance indicates that the feature is important, while a negligible change suggests it has little influence. This approach provides a straightforward way to interpret the contribution of each feature in the RF model, helping to identify which features are most critical for making accurate diagnoses. Finally, the top 20 features were selected by the permutation importance strategy to build the ML model.

### 2.7 Statistical Analysis

Continuous variables were expressed mean ± standard deviation (SD) or median (interquartile range), depending on the distribution of the data, and were analysed by an independent t test or Mann–Whitney *U* test. The independent *t*‐test was applied when the data followed a normal distribution to compare the means between two groups, while the Mann–Whitney *U* test was used for non‐normally distributed data as it is a non‐parametric alternative that compares medians. Categorical variables were presented as absolute numbers (*n*) and relative frequencies (%) and were analysed by the chi‐square test. ML classification models were built using Python software, and accuracy, precision, sensitivity, specificity, F1 score and area under the curve (AUC) (95% confidence interval [CI]) were presented. Differences with *p* < 0.05 were considered statistically significant. The data were analysed using SPSS version 18 (IBM Corp., Armonk, N.Y., USA).

### 2.8 Ethics Approval and Consent to Participate

The study was approved by the ethics committee of the Fasa University of Medical Sciences (approval number: IR.FUMS.REC.1401.240) and adhered to Helsinki guidelines. Furthermore, all subjects provided written informed consent before participating. The authors have no access to information that could identify individual participants during or after data collection.

## 3 Results

The participants with the mean ± SD of age of 48.63 ± 9.57 years and a male‐to‐female ratio of 0.8:1 reported that 9.8% had osteoporosis. Table 1 presents the top 20 features of all participants and a comparative analysis of key demographic and clinical characteristics of the study population, split between non‐osteoporosis and osteoporosis groups. Understanding these characteristics is essential for assessing baseline disparities and providing context for how osteoporosis correlates with various sociodemographic and health‐related factors.

**TABLE 1:** The top 20 features of all study participants.

	Total (*N* = 10,108)	Non‐osteoporosis (*N* = 9116)	Osteoporosis (*N* = 992)	*p*
Age	48.63 ± 9.57	47.78 ± 9.30	56.45 ± 8.36	**< 0.01**
*Sex* ^a^				
Male	4565 (45.2)	4451 (48.8)	114 (11.5)	**< 0.01**
Female	5543 (54.8)	4665 (51.2)	878 (88.5)	
BMI, kg/m^2^ ^a^	25.65 ± 4.82	25.57 ± 4.80	26.47 ± 4.95	**< 0.01**
*Marital status*				
Single	372 (3.7)	356 (3.9)	16 (1.6)	**< 0.01**
Married	8991 (88.9)	8179 (89.7)	812 (81.9)	
Widowed	643 (6.4)	489 (5.4)	154 (15.5)	
Divorced	102 (1.0)	92 (1.0)	10 (1.0)	
Having motorcycle	6829 (67.6)	6247 (68.5)	582 (58.7)	**< 0.01**
*Socioeconomic status*				
Low	2531 (25.0)	2219 (24.3)	312 (31.5)	**< 0.01**
Middle	5047 (50.0)	4531 (49.7)	516 (52.0)	
High	2530 (25.0)	2366 (26.0)	164 (16.5)	
*Past medical history*				
Joint pain	4203 (41.6)	3473 (38.1)	730 (73.6)	**< 0.01**
Cardiovascular diseases	1095 (10.8)	875 (9.6)	220 (22.2)	**< 0.01**
Chronic headaches	1569 (15.5)	1353 (14.8)	216 (21.8)	**< 0.01**
Family history of epilepsy	610 (6.0)	539 (5.9)	71 (7.2)	> 0.05
*Medicine intake*				
Calcium intake, mg/day	1418.65 ± 683.26	1436.45 ± 686.16	1255.10 ± 633.34	**< 0.01**
Vitamin D intake, IU	50.31 ± 41.62	50.49 ± 41.29	48.59 ± 44.47	> 0.05
Dosage use of calcium pills	0.02	0.07	0.01	**< 0.05**
Dosage use of calcium‐vitamin D pills	0.01	0.06	0.01	**< 0.01**
*Laboratory findings*				
Red blood cell count, ×10^3^/mm^3^	4.96 ± 0.54	4.97 ± 0.56	4.85 ± 0.51	**< 0.01**
Platelet count, ×10^3^/mm^3^	274.32 ± 71.77	273.47 ± 71.66	282.10 ± 72.38	**< 0.01**
MCV, fL	85.07 ± 7.60	85.09 ± 7.61	84.82 ± 7.55	> 0.05
Alkaline phosphatase, IU/L	209.36 ± 71.04	207.99 ± 71.20	221.95 ± 68.25	**< 0.01**
Gamma‐glutamyl transpeptidase, IU/L	22.82 ± 21.09	22.92 ± 21.67	21.89 ± 14.62	0.14
Aspartate aminotransferase, IU/L	22.56 ± 8.46	22.58 ± 8.55	22.34 ± 7.58	> 0.05
Urine protein	490 (4.8)	448 (4.9)	42 (4.2)	> 0.05
Urine ketone bodies	647 (6.4)	584 (6.4)	63 (6.4)	> 0.05

*Note:* Data were presented as mean ± SD and number (%). Statistical analyses such as the independent samples test or chi‐square were used. Values in bold indicates statistical significance of *P* < 0.05.

Abbreviations: BMI, body mass index; MCV, mean corpuscular volume.

^a^ Sex and BMI are the only demographic variables in the study and not among the top 20 variables.

The prevalence of osteoporosis was significantly higher in older married females with a higher BMI, middle socioeconomic status and history of joint pain, cardiovascular diseases and chronic headaches. Additionally, it is common among participants to consume less calcium and to take fewer calcium‐vitamin D pills. According to laboratory findings, osteoporosis is associated with higher platelet counts, alkaline phosphatase levels and a lower red blood cell (RBC) count. The variables, including the family history of epilepsy, vitamin D intake, mean corpuscular volume (MCV), gamma‐glutamyl transpeptidase, aspartate aminotransferase, urine protein and urine ketone bodies, did not have a significant association with having or not having osteoporosis.

### 3.1 Model Performance

All the top‐20 variables were employed in the ML algorithms, and the performance of the diagnosis models on the test data set for all ML techniques is shown in Table 2. Table 2 showcases the performance metrics of various diagnosis models, evaluating each model's accuracy, precision, sensitivity, specificity, F1 score and AUC. These results highlight the strengths and limitations of each model in diagnosing osteoporosis risk, guiding readers in understanding the comparative effectiveness and potential applications of each algorithm in this context.

**TABLE 2:** Performance of the diagnosis models.

Models	Accuracy (95% CI)	Precision (95% CI)	Sensitivity (95% CI)	Specificity (95% CI)	F1‐score (95% CI)	AUC (95% CI)
LR	0.77 (0.73–0.81)	0.24 (0.21–0.27)	0.66 (0.62–0.70)	0.78 (0.74–0.82)	0.36 (0.32–0.40)	0.78 (0.74–0.82)
DT	0.79 (0.75–0.83)	0.23 (0.20–0.26)	0.55 (0.51–0.59)	0.81 (0.77–0.85)	0.34 (0.30–0.38)	0.76 (0.72–0.80)
SVC	0.64 (0.60–0.68)	0.15 (0.13–0.17)	0.67 (0.63–0.71)	0.64 (0.60–0.68)	0.27 (0.24–0.30)	0.71 (0.66–0.75)
RF	0.77 (0.74–0.90)	0.31 (0.28–0.34)	0.42 (0.38–0.46)	0.90 (0.87–0.93)	0.36 (0.32–0.40)	0.78 (0.75–0.82)
LDA	0.76 (0.72–0.80)	0.24 (0.21–0.27)	0.67 (0.63–0.71)	0.77 (0.73–0.81)	0.35 (0.31–0.39)	0.78 (0.74–0.82)
KNN	0.59 (0.55–0.63)	0.14 (0.12–0.16)	0.61 (0.57–0.65)	0.58 (0.54–0.62)	0.22 (0.19–0.25)	0.63 (0.59–0.67)
XGB	0.79 (0.75–0.83)	0.25 (0.22–0.28)	0.58 (0.54–0.62)	0.81 (0.77–0.85)	0.36 (0.32–0.40)	0.78 (0.74–0.82)
Baseline LR	0.82 (0.78–0.86)	0.24 (0.21–0.27)	0.37 (0.33–0.41)	0.87 (0.84–0.9)	0.29 (0.26–0.32)	0.72 (0.68–0.76)

Abbreviations: CI, confidence interval; DT, decision tree; KNN, K nearest neighbours; LDA, linear discriminant analysis; LR, logistic regression; RF, random forest; SVC, support vector classifier; XGB, extreme gradient boosting.

The performance of eight ML models for diagnosing osteoporosis was evaluated, with notable results across various metrics. The highest accuracy was achieved by baseline LR at 82% and then DT and XGB, both at 79%, followed by LR and RF at 77%. In terms of precision, RF led with 31%, followed by XGB at 25%. Sensitivity was highest in LDA and SVC at 67%, with LR close behind at 66%. RF demonstrated the highest specificity at 90%, followed by baseline LR at 87% and XGB and DT, each at 81%. The F1 score was highest for XGB, RF and LR at 36%, with LDA at 35%. Lastly, the AUC was highest for XGB, LDA, RF and LR at 78%, followed by DT at 76%. As expected, this baseline model exhibited poorer performance across the prioritised metrics (AUC, F1 score and sensitivity) compared to the more advanced models. This further highlights the added value of employing more complex ML techniques such as XGBoost and Random Forest for this task.

Overall, the XGB model was the best‐performing model, achieving the highest AUC and F1 score, which were the primary criteria for selecting the top model. Additionally, XGB showed strong performance in precision and specificity. The RF model also performed well, with high scores in precision, specificity and AUC, and its accuracy was among the top performers. However, based on our prioritisation of AUC, F1 score and sensitivity, XGB was selected as the best model for diagnosing osteoporosis risk. The AUC, F1 score and sensitivity as the primary evaluation metrics were prioritised because the focus of this study is on accurately identifying individuals at risk of osteoporosis, which corresponds to correctly predicting positive outcomes. Sensitivity was emphasised to minimise false negatives, ensuring that high‐risk individuals are not missed. The F1 score was selected to balance precision and recall, both of which are critical in a medical diagnostic context where both over‐diagnosis and under‐diagnosis can have significant consequences. AUC was chosen to assess the model's overall discriminatory power across all classification thresholds, providing a comprehensive evaluation of performance.

### 3.2 Feature Importance

**FIGURE 1:**
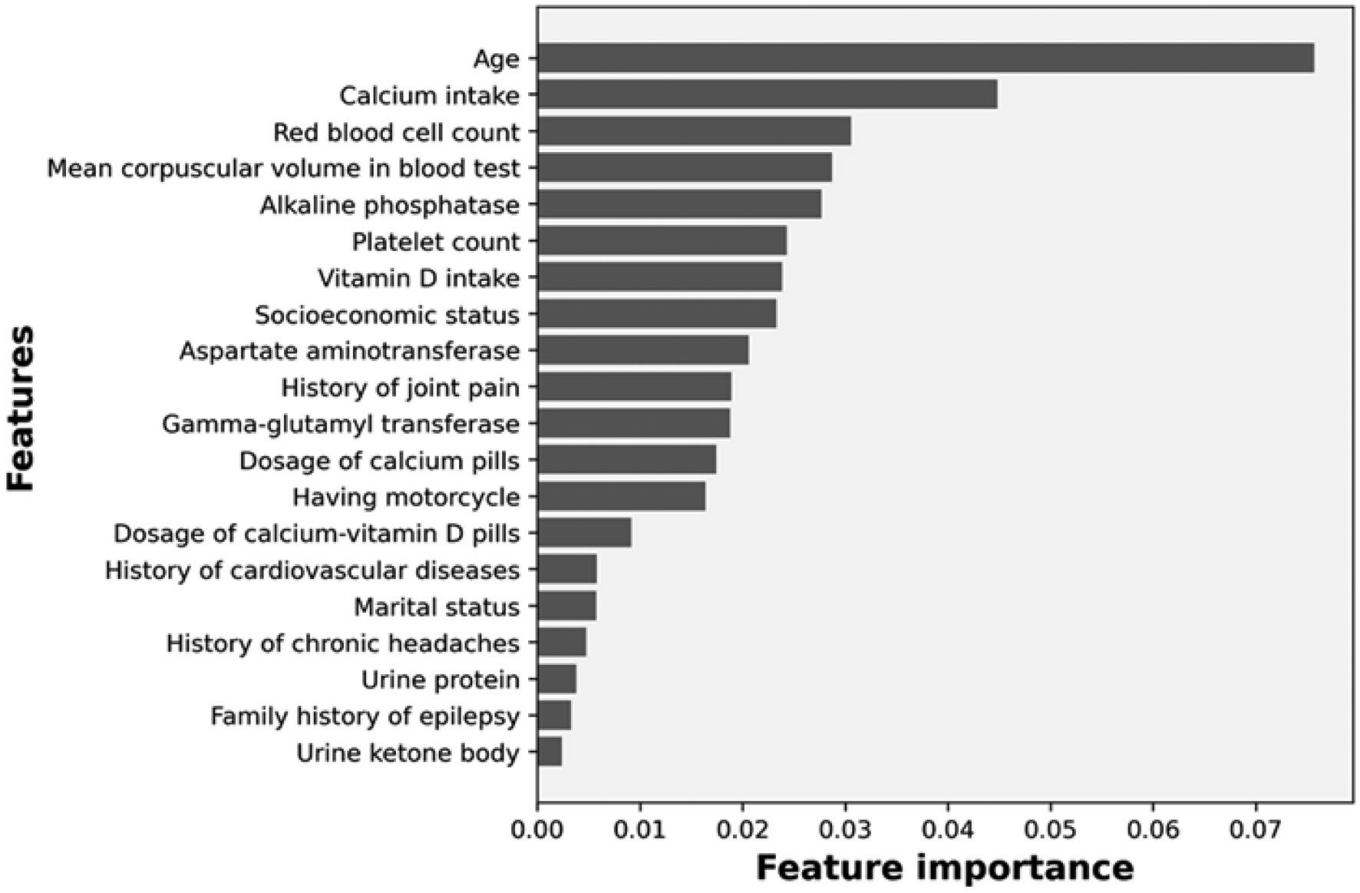
Feature importance of top‐20 diagnosers.

Feature importance for the XGB model is given in Figure 1. Age, calcium intake and RBC count were the three key variables for diagnosing osteoporosis.

**FIGURE 2:**
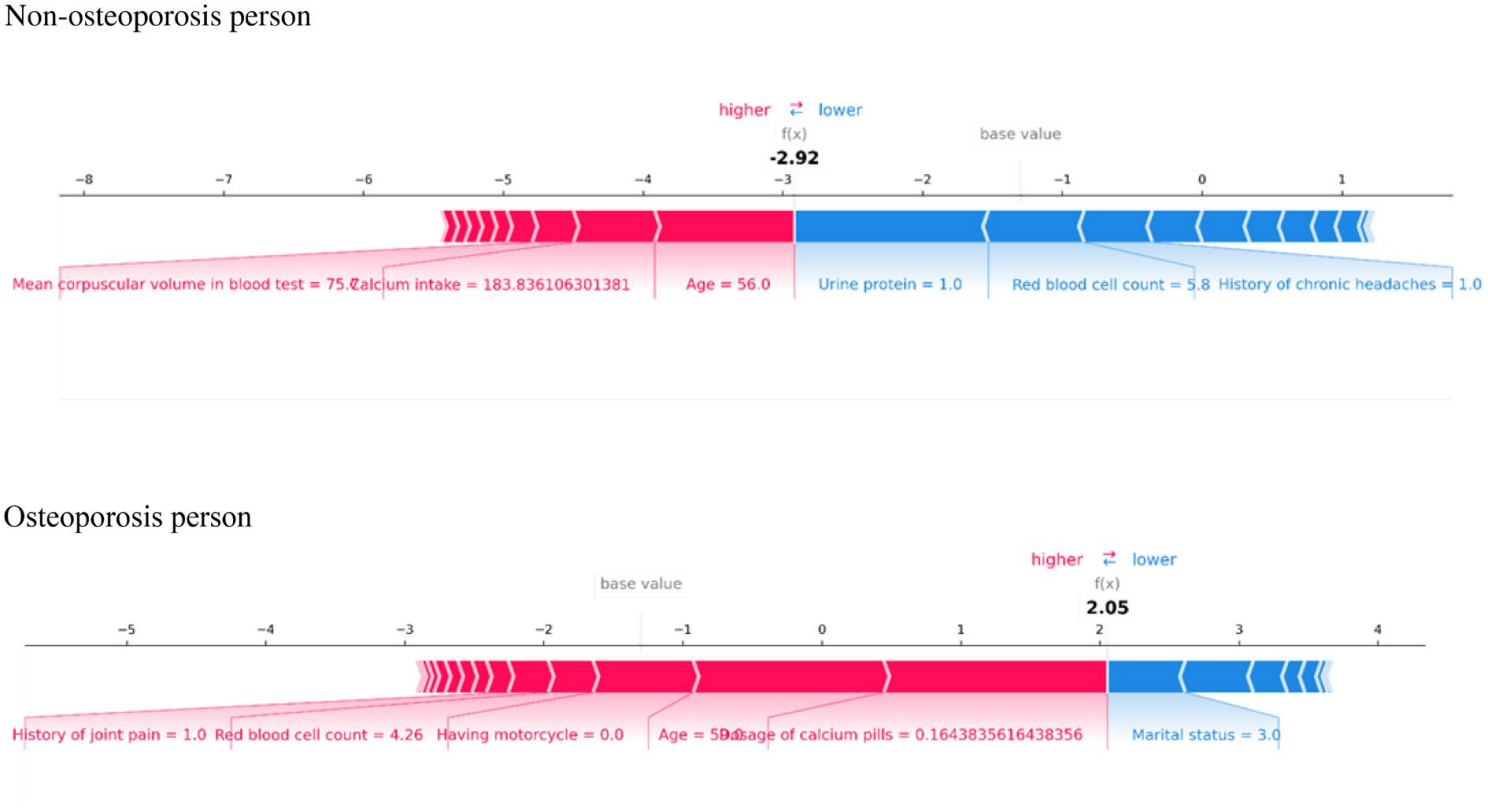
Shapley additive explanation force plot for two selected patients.

Figure 2 shows individual SHAP force plots for patients who are (A) non‐osteoporosis people and (B) osteoporosis people.

SHAP values display the characteristics associated with the diagnosis of individual patients, as well as the contribution of each characteristic to the diagnosis of osteoporosis. The bold numbers are the possible diagnosed values (*f*(*x*)), while the base values are the values diagnosed without giving input to the model. The *f*(*x*) is the odds ratio of each observation. The red features (on the left) indicate features that increase the risk of osteoporosis, and blue features indicate features that decrease the risk of osteoporosis. The length of the arrows helps visualise the magnitude of the effect on the diagnosis. The longer the flash, the bigger the effect.

Among the osteoporosis participants with *f*(*x*) of 2.05, the dosage of calcium pills of 0.16, the age of 59 years, not having a motorcycle, RBC counts of 4.26 × 10^3^/mm^3^ and the history of joint pain are the features that increase the risk of osteoporosis in order of most effect to least effect and the marital status of a widow decreases the risk of osteoporosis.

Among the non‐osteoporosis participants with *f*(*x*) of −2.92, in order of most effect to least effect, the age of 56 years, calcium intake of 183.8 and the MCV of 75.7 fL increase the risk and urine protein excretion, RBC counts of 5.8 × 10^3^/mm^3^ and history of chronic headache decrease the risk of osteoporosis.

## 4 Discussion

To develop an ML model for osteoporosis diagnosis, we analysed 20 factors, including demographic information, laboratory tests and clinical examinations, collected from 10,108 individuals from the FACS. After evaluating the F1 score, AUC and sensitivity, we have determined that the XGB model outperforms other models in this domain. According to the obtained results, the baseline logistic regression exhibited weaker performance in important metrics, including AUC, F1 score and sensitivity, compared to more complex models.

The XGB model identified age, calcium intake, RBC count, MCV and alkaline phosphatase levels as the most important variables for diagnosis. By examining and measuring the mentioned characteristics in individuals, it is possible to identify those at risk of developing the disease or those in its silent stages. This way, more comprehensive and specialised measures for prevention, determining the stage and severity of disease progression, and timely treatment can be allocated to these selected individuals.

Although the XGB model demonstrated the highest AUC of 0.78 (95% CI: 0.74–0.82), it exhibited a relatively low precision of 25%. This discrepancy suggests a trade‐off between the model's ability to correctly identify true positives (high sensitivity) and its tendency to generate false positives (low precision). While AUC reflects the model's overall ability to discriminate between classes, it does not capture this imbalance between precision and recall. The low precision indicates that, despite the model's strong overall classification ability, it may produce a higher number of false positives. This is a common challenge in imbalanced datasets where the minority class is oversampled, as in this study. Future work could explore ways to improve this balance, such as tuning the decision threshold or applying more advanced resampling techniques.

Aging disrupts the balance by disrupting the rate of osteoblasts and increasing the breakdown and absorption of bone tissue, which in turn impacts the incidence of osteoporosis [15]. Many studies have been conducted on the effect of diet and the importance of calcium intake in populations at high risk of osteoporosis. In a study conducted in Japan in 2018, the impact of daily consumption of calcium supplements, even at a low dose, on reducing the rate of breakdown and absorption of spinal column bones was assessed [16]. Based on a systematic review conducted in 2020, it was found that only consuming dairy rich in vitamin D and calcium has a significant impact on increasing BMD [17]. In 2020, a study on the relationship between vertebral fractures and calcium intake in the Chinese cohort population showed that a diet high in calcium significantly reduces the risk of vertebral fractures [18]. As indicated in this study, age has a significant effect on osteoporosis and is the most important feature to diagnose it.

In many articles, the effect of haematopoietic stem cell transplantation on increasing the risk of osteoporosis and fragility fractures has been analysed [19, 20]. Clinical evidence suggests that anaemia resulting from ageing contributes to an increased risk of bone fractures [21]. In 2011, a study was conducted on a population of menopausal Korean women, revealing an interesting correlation between low BMD and the prevalence of osteoporosis and low blood cell counts, including RBCs and white blood cells [22]. Also, the high levels of red cell distribution width and anaemia, together, increase the risk of clinical bone fractures [23], and there is an inverse relationship between BMD and MCV [24]. In this study, it is mentioned that, besides the importance of RBC counts as a diagnostic feature of osteoporosis, it is notable to mention that the RBC counts of 4.26 × 10^3^/mm^3^ and its MCV of 75.7 fL can increase the risk of osteoporosis. It was amazing that with the increase of the RBC counts to 5.8 × 10^3^/mm^3^, it decreased the risk of osteoporosis among non‐osteoporosis participants.

Alkaline phosphatase changes in bones can indicate alterations in bone balance. There is an inverse relationship between the level of serum alkaline phosphatase and lumbar BMD, regardless of gender and race. It was believed that measuring alkaline phosphatase could be used as a factor for early diagnosis of osteoporosis [7, 25]. In this study, it is shown that alkaline phosphatase is a significant association between osteoporosis and non‐osteoporosis participants, and it is the 5th important factor in the diagnosis of osteoporosis.

Based on our findings in this study and previous studies, we believe that an application can be designed that is available online to everyone. By taking into account the risk factors of each individual, this app can calculate the risk of developing osteoporosis. Such a tool allows for screening and identifying individuals at risk before the onset of severe and fatal complications. In the absence of advanced diagnostic tools and the lack of funds, facilities and necessary human resources, particularly in developing countries with weak economies, the presence of such tools enables individuals at a higher risk of contracting the disease to distinguish themselves from others, conduct investigations, introduce more advanced tools and provide necessary recommendations to control the disease's risk factors. With the early diagnosis of people with or at high risk of osteoporosis, the morbidity and mortality of this disease will be under control, and a heavy burden will be removed from the healthcare systems of countries and, of course, individuals.

### 4.1 Limitations and Strengths

A notable constraint of this study is its dependence on self‐reported data for most non‐communicable disorders, such as osteoporosis, which could result in mistakes. The self‐reported data entered into this study can introduce error in the study population and consequently reduce the accuracy and precision of the results obtained from the ML models. While the self‐reported data is verified by cohort physicians by examining drug consumption records, current diseases, medical histories and patient‐provided medical records, there is still a possibility of a small underestimation in apparently healthy individuals. Although ML models were utilised for diagnosis, the middling performance metrics suggest that there is scope for enhancing diagnosis accuracy. Although SMOTE was employed to address the class imbalance by generating synthetic data points for the minority class, it has certain limitations, particularly in clinical datasets.

The synthetic instances generated by SMOTE may introduce artificial patterns or artefacts that do not accurately represent real‐world clinical variability. In medical datasets, small data nuances can be important, and SMOTE might fail to capture these, potentially leading to unrealistic or biologically implausible synthetic samples. Additionally, there is a risk of overfitting if the synthetic points are overly similar to the existing minority class points, which could compromise the model's generalisability to new data. However, to mitigate these risks, SMOTE was applied only to the training data, while the test data remained untouched and unseen, simulating real‐world conditions for model evaluation.

This ensured that the model's performance was evaluated on real, unseen data and not influenced by the synthetic data used in training. The use of ‘calcium intake’ instead of serum calcium level’ in this study may not fully reflect the biological effects of calcium on bone health. Calcium supplementation may confuse osteoporosis patients. Patients with osteoporosis may use more calcium supplements, which may affect the observed link between calcium consumption and risk in this sample. Patients with osteoporosis and higher calcium supplement usage may influence algorithmic accuracy. Calcium consumption may appear to be a weaker osteoporosis predictor, especially in non‐supplementing adults. Future investigations should use serum calcium measures as a more reliable physiological biomarker of calcium status. Separating dietary calcium intake from supplementation may assist osteoporosis patients to avoid supplementation bias. Finally, the conclusions of the study are restricted to the specific demographic and geographic range of Fasa's rural population and may not be applicable to other communities.

An admirable aspect of this study is its utilisation of a substantial and thoroughly described group of individuals from the FACS, consisting of more than 10,000 participants. This ensures the availability of a strong and reliable dataset for thorough examination. The longitudinal strategy, incorporating many follow‐up periods, enables the evaluation of temporal changes and augments the dependability of the results. Utilising advanced ML approaches, such as hyperparameter tweaking and cross‐validation, improves the reliability and diagnostic accuracy of the models employed. Moreover, employing permutation importance for feature selection offers a lucid comprehension of the most relevant variables in diagnosing osteoporosis.

## 5 Conclusion

This study found the most successful ML algorithm, XGB, to diagnose osteoporosis. The results of the traditional baseline LR model, in which feature selection methods are not used, have less value and credibility compared to the results obtained from more advanced models. Additionally, this study determined age, calcium intake, RBC count, MCV and alkaline phosphatase levels as the most important variables for diagnosis of osteoporosis. The key point is that using tailored and preventive approaches that target the identified risk factors can improve the management and prevention of osteoporosis. We believe that an easily accessible algorithm can be developed based on our study and future research and investigations, which assess individual risk factors for osteoporosis. In the absence of advanced diagnostic tools and with the lack of financial resources, facilities and necessary personnel, especially in developing countries with weak economies, having such tools would help distinguish individuals at high risk of the disease from others, leading to further evaluations for them. By facilitating early detection, it could help control complications and mortality associated with the disease, thus reducing the burden on healthcare systems and benefiting individuals.

Subsequent studies should focus on confirming these results in various populations and investigating the incorporation of supplementary factors to enhance the precision of the model. Furthermore, it is necessary to conduct prospective research in order to establish a cause‐and‐effect relationship and to further improve diagnosis models. Subsequent investigations should focus on confirming these results in various demographics and investigating the incorporation of supplementary factors and larger sample sizes to enhance the precision of the model. Furthermore, it is necessary to conduct further studies in order to determine causality and enhance the accuracy of diagnosis models. We recommend conducting longitudinal studies and follow‐ups on a wider statistical population to strengthen the diagnosis algorithms and collecting more specialised data related to osteoporosis from advanced research and treatment institutions for this disease. We also suggest using other ML algorithms to compare the results.

## Author Contributions

All authors made significant contributions to the design of the study, analysing the data and drafting the manuscript. S.T., O.K., B.P., A.A., S.D.A. and R.T. contributed to drafting the article or revising it. O.K., S.T., S.D.A. and R.T. approved the revised version to be submitted. All authors have read and accepted the manuscript.

## Ethics Statement

The Fasa University of Medical Sciences approved the study protocol (IR.FUMS.REC.1401.240). The research procedures were conducted in accordance with all relevant guidelines and regulations. Before participating, individuals were asked to give written informed consent, which was reviewed and approved by the research ethics committee. Participant personal information was collected from the system, ensuring that the identities of all individuals remained anonymous.

## Consent

The authors have nothing to report.

## Conflicts of Interest

The authors declare no conflicts of interest.

## Supporting information


Table S1.

## Data Availability

The data that support the findings of this study are available from the corresponding author upon reasonable request.
