# Supplementary material for: Diagnosis Osteoporosis Risk: Using Machine Learning Algorithms Among Fasa Adults Cohort Study (FACS)
Source: Endocrinol Diabetes Metab. 2025 Jan 6;8(1):e70023. doi: 10.1002/edm2.70023 (PMC11702405; doi:10.1002/edm2.70023)
Supplement: Supplementary file 1 — Table S1. [file EDM2-8-e70023-s001.docx]

**Diagnosis Osteoporosis Risk: Using Machine Learning Algorithms among Fasa Adults Cohort Study (FACS)**

Supplementary appendix

**Supplementary Table 1. Optimal hyper-parameters after hyper-parameter tuning with grid search method**

| Logistic Regression | C: 10, solver: 'newton-cg', multi_class: 'auto', penalty: 'l2' |
| --- | --- |
| Decision Tree Classifier | criterion: 'entropy', max_depth: 4 |
| Support Vector Classifier | C: 1, gamma: 'scale', kernel: 'rbf', probability: True |
| Random Forest Classifier | max_features: 'sqrt', n_estimators: 1000 |
| K Neighbors Classifier | metric: 'manhattan', n_neighbors: 19, weights: 'distance' |
| Linear Discriminant Analysis | shrinkage: 'auto', solver: 'lsqr', tol: 0.0001 |
| XGBoost Classifier | n_estimators: 180, colsample_bytree: 0.5, gamma: 1, max_depth: 1, min_child_weight: 1, reg_alpha: 1, reg_lambda: 1 |
